# Clinical Translation and Implementation of a Bioartificial Pancreas Therapy: A Qualitative Study Exploring the Perspectives of People With Type 1 Diabetes

**DOI:** 10.1097/TXD.0000000000001711

**Published:** 2024-09-25

**Authors:** Dide de Jongh, Silke Lapré, Behiye Özcan, Robert Zietse, Eline M. Bunnik, Emma K. Massey, the VANGUARD consortium

**Affiliations:** 1 Department of Internal Medicine, Erasmus MC Transplant Institute, University Medical Centre Rotterdam, Rotterdam, The Netherlands.; 2 Department of Medical Ethics, Philosophy and History of Medicine, Erasmus MC, University Medical Centre Rotterdam, Rotterdam, The Netherlands.; 3 Department of Internal Medicine, Erasmus MC, University Medical Center Rotterdam, Rotterdam, The Netherlands.; Department of Surgery, University of Geneva, Geneva, Switzerland; Department of Surgery, University of Geneva, Geneva, Switzerland; Department of Surgery, University of Geneva, Geneva, Switzerland; Department of Surgery, University of Geneva, Geneva, Switzerland; Department of Surgery, University of Geneva, Geneva, Switzerland; Diabetes Centre—Campus Innenstadt, Medizinische Klinik und Poliklinik IV, Klinikum der Ludwig-Maximilians-Universität München, Germany; Diabetes Centre—Campus Innenstadt, Medizinische Klinik und Poliklinik IV, Klinikum der Ludwig-Maximilians-Universität München, Germany; Diabetes Centre—Campus Innenstadt, Medizinische Klinik und Poliklinik IV, Klinikum der Ludwig-Maximilians-Universität München, Germany; Diabetes Centre—Campus Innenstadt, Medizinische Klinik und Poliklinik IV, Klinikum der Ludwig-Maximilians-Universität München, Germany; Diabetes Centre—Campus Innenstadt, Medizinische Klinik und Poliklinik IV, Klinikum der Ludwig-Maximilians-Universität München, Germany; Diabetes Centre—Campus Innenstadt, Medizinische Klinik und Poliklinik IV, Klinikum der Ludwig-Maximilians-Universität München, Germany; Department of Health Sciences, University of Piemonte Orientale, Novara, Italy; Department of Health Sciences, University of Piemonte Orientale, Novara, Italy; Department of Health Sciences, University of Piemonte Orientale, Novara, Italy; Department of Health Sciences, University of Piemonte Orientale, Novara, Italy; Department of Health Sciences, University of Piemonte Orientale, Novara, Italy; IRCCS Ospedale San Raffaele, Diabetes Research Institute, Milano, Italy; IRCCS Ospedale San Raffaele, Diabetes Research Institute, Milano, Italy; IRCCS Ospedale San Raffaele, Diabetes Research Institute, Milano, Italy; IRCCS Ospedale San Raffaele, Diabetes Research Institute, Milano, Italy; IRCCS Ospedale San Raffaele, Diabetes Research Institute, Milano, Italy; Department of Transplantation, Nephrology and Clinical Immunology, Lyon Claude Bernard University, Lyon, France; Department of Transplantation, Nephrology and Clinical Immunology, Lyon Claude Bernard University, Lyon, France; European Society for Organ Transplantation, Padova, Italy; European Society for Organ Transplantation, Padova, Italy; European Society for Organ Transplantation, Padova, Italy; Kugelmeiers AG, Erlenbach, Switzerland; Kugelmeiers AG, Erlenbach, Switzerland; Kugelmeiers AG, Erlenbach, Switzerland; Accelopment Switzerland Ltd; Accelopment Switzerland Ltd; Accelopment Switzerland Ltd

## Abstract

**Background.:**

The development of a hybrid beta-cell replacement approach, referred to as a personalized, transplantable bioartificial pancreas (BAP), holds promise to treat type 1 diabetes (T1D). This interview study aimed to explore patients’ expectations, needs, concerns, and considerations when considering to undergo a BAP transplantation.

**Research Design and Methods.:**

Semistructured interviews were conducted with 24 participants diagnosed with T1D. Data collection stopped once data saturation was reached. Audio recordings of the interviews were transcribed verbatim. The interviews were independently analyzed by 2 researchers. A qualitative content analysis using an inductive approach was used.

**Results.:**

Three main themes emerged as follow: (1) hoped-for benefits, (2) concerns and decision-making considerations, and (3) procedural aspects. First, the participants expected benefits across medical, psychological, and social domains. Over these 3 domains, 9 subthemes were identified, including improved clinical outcomes, a cure for diabetes, more headspace, emotional relief, a shift in responsibility, protection of privacy, improved flexibility in daily life, less visible diseases, and improved relationships with others. Second, concerns and considerations about undergoing a BAP transplant comprised adverse events, the functionality of the BAP, the surgery procedure, the biological materials used, the transplant location, and the intrusiveness associated with follow-up care. Finally, procedural considerations included equitable access, patient prioritization, and trust and control.

**Conclusions.:**

Incorporating insights from this study into the clinical development and implementation of the BAP is crucial to ensure alignment of the product and procedures with the needs and expectations of people with T1D.

The field of regenerative medicine is developing a hybrid beta-cell replacement approach for type 1 diabetes (T1D), referred to as the “bioartificial pancreas” (BAP). If successfully developed, this approach could have a revolutionary effect on the global management of T1D. Exploring the perspectives of people with T1D on this technology is needed to facilitate ethically responsible clinical translation of this therapy into care.

The introduction of advanced technical devices such as hybrid closed loop (HCL) systems has improved glucose regulation^1^ and favorable psychosocial outcomes for people with T1D, including reduced levels of stress and anxiety.^2^ Despite the benefits, handling these devices can be challenging for people with T1D, especially those with poor health literacy skills.^3^ One issue is the necessity for continual manual adjustment of parameters according to calorie intake and energy expenditure, which can be experienced as burdensome^3^ and has been associated with diabetes-related distress. In addition, suboptimal glycemic control may still occur, which in turn increases the risk of long-term neurologic, microvascular, and macrovascular complications.^4,5^

Allogeneic pancreas or islets of Langerhans transplantation are alternative approaches to treat people with T1D, which could return the recipient to a state of euglycemia, reduce glycemic variability, restore hypoglycemic awareness, and resolve severe hypoglycemia.^6^ However, only a small number of individuals with T1D with severe complications, such as hypoglycemic unawareness, suboptimal blood glucose control despite maximal medical input, and kidney failure, undergo transplantation.^7^ This is because of the shortage of organs derived from deceased donors and the need for lifelong immunosuppressive medication.^7^ Consequently, there is a global drive to develop alternative therapies to overcome the limitations associated with current treatment options.

Significant progress has been made in the development of a BAP.^8,9^ This advanced therapeutic medicinal product approach, designed and assembled in laboratories, can be composed of metabolically active cellular components combined with synthetic (i.e. non-biological) medical devices. A device is used to protect and hold insulin-secreting cells in position and increase the long-term capacity of these cells. Insulin-secreting cells can potentially be obtained from deceased donors, xenogeneic cells, or genetically modified allogeneic stem cells. These cells could be used in conjunction with biological materials derived from various sources, such as amniotic epithelial cells from placentas, to safeguard the BAP against local immune responses, thereby avoiding the need for immunosuppression. This approach holds promise and may offer an alternative to a larger group of people with T1D, particularly if renewable sources for islet cells can be found.

Although advancements in BAP development show promise,^10,11^ research participants enrolled in early-phase clinical trials are inevitably exposed to risks. It is essential that harms are minimized to justify the enrollment of human research participants.^12^ This requirement is challenging to meet because of the surgical procedures involved in transplanting an advanced therapeutic medicinal product into human recipients.^13^ Incorporating perspectives of potential users into design and technology considerations will help safeguard ethically responsible and acceptable translation of such investigational therapies from bench to bedside. This is the first qualitative study evaluating the perspectives of people with T1D regarding the BAP to understand their expectations, needs, concerns, and potential considerations they would consider when deciding to undergo BAP transplantation.

## MATERIALS AND METHODS

### Study Design

This is a cross-sectional, single-center interview study. A qualitative research approach was used to gain an in-depth understanding of the perspectives of people with T1D.

### Sampling and Recruitment

We included individuals with T1D treated in an academic hospital, representing a sample that may be eligible for clinical trials or early adoption of the BAP. Academic hospitals generally serve adults with T1D who have diverse diabetes-related complications and require more complex care compared with those treated in regional nonacademic hospitals. In our center, we do not conduct islet or pancreas transplantation therefore transplant candidates or recipients were not included. The participants were largely recruited through consecutive sampling at the outpatient diabetes clinic at the Erasmus MC, University Medical Centre Rotterdam, in the Netherlands. However, this sampling method led to the inclusion of mainly older participants. Therefore, we included a purposive sample of younger participants (20–39 y) to ensure the inclusion of their perspectives. The inclusion criteria were that the participants were older than 18 y and could speak and understand Dutch. Participants recruited in the outpatient clinic were approached face-to-face by their treating physician (B.O.). The purposive sample of younger people with T1D was recruited via the network of the research team.

### Data Collection

The semistructured interviews were conducted in Dutch between September 2022 and March 2023 by D.J. (cisgender woman, MSc). Before the start of the interviews, a guide (**Supplemental Material 1, SDC,** http://links.lww.com/TXD/A700) was developed by researcher D.J. and discussed with the multidisciplinary research team E.K.M., E.M.B., B.O., and E.M.B. (psychologist, medical ethicist, endocrinologist, and project leader of the VANGUARD consortium). The concept of a BAP was new to all participants. Therefore, the interviewer (D.J.) started the interviews with a brief standardized PowerPoint presentation providing information about the BAP (**Supplemental Material 2, SDC,** http://links.lww.com/TXD/A700). In summary, she explained that a transplantable, personalized BAP therapy would ideally not require immunosuppressive medication and could consist of biological material derived from various sources, such as deceased donors, genetically modified donated placentas, pigs, and patient’s own cells. The ultimate goal is to transplant this therapy into patients, potentially making them insulin independent. No prior information was given about the possibility or need for a continuous glucose monitor (CGM) in combination with the BAP.

The participants were informed of the background and occupation of D.J. No personal relationships were established between D.J. and the participants before data collection. All interviews took approximately 1 h and were audiotaped. The interviews were transcribed verbatim using the transcription program Amberscript. D.J. checked these transcripts and manually anonymized them. The participants did not provide feedback on the transcripts and findings. Additionally, sociodemographic and medical information (eg, current treatment, age, sex) was collected from the medical files and verified by the participants. Our methods are reported in accordance with the consolidated criteria for reporting qualitative research.^14^

### Data Analysis

The transcripts were independently analyzed by 2 researchers (D.J. and S.L.) using the data analysis software NVIVO version 12. An inductive approach based on the general principles of grounded theory was used.^15^ The transcripts were analyzed thematically, which involved line-by-line coding; similar codes were combined and then grouped together into themes and subthemes. Coders cross-compared and reviewed their coded transcripts weekly to identify similarities and differences. A coding framework was developed to capture defined themes and subthemes.^16^ This coding framework was regularly discussed and refined with senior researchers experienced in qualitative research (E.K.M. and E.M.B.) until agreement. After analyzing 17 interview transcripts, no new codes were added to the coding framework. Seven interviews were conducted to verify whether data saturation was achieved.

### Ethical Approval

The research proposal was submitted for review by the Research Ethics Review Committee of Erasmus MC, and a waiver was granted (MEC-2022-0568).

## RESULTS

### Study Participants

A total of 24 persons with T1D consented to participate (50% women). Four of the 24 participants were recruited by purposive sampling, and none of the participants dropped out. Most interviews (n = 19) were undertaken live at participants’ homes or the Erasmus MC and 5 interviews were conducted online. In total, 20 participants used a CGM, 10 of whom used an HCL. Hemoglobin A1c (%) levels ranged from 5.2% to 9.7% (36–83 mmol/mol), with an average target level of approximately 7.2% (55 mmol/mol).^17^ The time in range measured via CGM ranged from 34% to 83%, with a target value of >70%.^18^ In total, 10 participants had diabetes-related complications, including macrovascular complications, polyneuropathy, diabetic retinopathy, diabetic nephropathy, limb amputations, and cataracts. Thirteen participants had hypoglycemic unawareness. Table 1 provides an overview of participant characteristics.

**TABLE 1.** Participants characteristics

Age range, y	N	%	Female, %	MDI, %	CSII, %	CGM, %	Years since diagnosis, mean (range)	Time in range (%), mean (range)^*a*^	HbA1c, %, mean (range)	HbA1c, mmol/mol mean (range)
20–39	9	37.5	55.5	45.5	55.5	88.9	16.3 (7–28)	71.5 (52–83)	7.1 (5.4–9.7)	53.9 (36–83)
40–59	7	29.2	42.9	57.1	42.9	57.1	26.0 (6–40)	72.0 (62–80)	7.4 (6.5–8.0)	57.0 (47–64)
60+	8	33.3	50.0	25.0	75	100	47.4(27–63)	66.6 (34–78)	7.2 (6.1–8.2)	54.8 (43–66)
Total	24	100	50	41.7	58.3	83.3	29.5 (6–63)	69.6 (34–83)	7.2 (5.4–9.7)	55.1 (36–83)

^*a*^Three participants with a continuous glucose monitor had no valid TIR data.

CGM, continuous glucose monitor; CSII, continuous subcutaneous insulin injection; hemoglobin A1c; MDI, multiple daily injection; TIR, time in range.

### Main Themes

The main topics discussed in the interviews were the benefits that people with T1D hoped a BAP might bring, their concerns and considerations in decision-making, and procedural considerations.

### Hoped for Benefits

Participants hoped that treatment with a BAP would benefit their lives across medical, psychological, and social domains. Participants mainly linked the benefits they hoped for to the challenges they have experienced with diabetes treatments. Over these 3 domains, 9 subthemes were identified: improved clinical outcomes, a cure for diabetes, more headspace, emotional relief, a shift in responsibility, protection of privacy, improved flexibility in daily life, disease being less visible, and improved relationships with others (Table 2).

**TABLE 2.** Hoped for benefits of treatment with the BAP

Domains	Subthemes	Quotations
Medical	Improved clinical outcomes A cure for diabetes	“…a huge advantage is of course that you are much more stable [in blood glucose rates] – at least as it works well. If you are much more stable, you will also feel more active in daily life and you will have a lower chance of developing long-term complications, then in my view you are a healthy person.”—participant 17 “… I would not see it [the BAP] as an alternative, but I would see it as the solution. A better solution than this [the BAP] does not exist.”—participant 6 “… [Y]ou still have it [T1D], but you wouldn’t notice it a whole lot anymore.”—participant 5
Psychological	More headspace Emotional relief A shift in responsibility Protection of privacy	“It’s because it [the BAP] is inside of your body you have little worry about it. Look, with those other systems it’s like: You must consider the batteries, the attachment points, changing the insulin, glucagon, you name it and that other thing [the BAP], that your body will do by itself.”—participant 12 “… You will indeed get a kind of your life back; you do not longer have to think about all the choices you have to make every day around food etc.”—participant 10. “Yeah, the problem is in those hypo’s and the hypers, that you are disrupted, and that you have to take that into account all the time, that that can happen and that you can be in the car with children, and you get a hypo…. That you endanger the lives of others. That’s the whole problem with that disease…. You would, I think … not have to be afraid in the car, or at other times.”—participant 5 “Then it would no longer be like: ‘you didn’t do that right’. No, it would be your little device [BAP] that is at fault.”—participant 7 “Perhaps there are still companies behind the production of modified cells. But the fewer they are, the more I would like that, because I have less confidence in that [manufactures] will protect my privacy. Especially the power that those large companies have. That is something I regularly worry about.”—participant 4
Social	Improved flexibility in daily life Disease being less visible Improved relationships with others	“… [S]ometimes I find myself in the situation that we are sitting at the table as a family and that someone says: Oh, I’m going to go for a work-out, are you joining, mom? And then I say: No, I can’t, because I have already injected insulin. Well and then you know on that point, if you would have the BAP, that you indeed can just say: Sure thing, I’ll do it. I’m going with you, because that seems very nice.”—participant 8 “… I think that for example for children it could have a social impact if they had a BAP…. If it doesn’t show that you have diabetes, then … individuals [with T1D] are not going to think they are being judged negatively.”—participant 1 “My marriage has been a real drama, because of the T1D. My wife had contemplated me that she will leave me, because of my fluctuating mood and the alarms of the CGM device going off during the night.”—participant 9

BAP, bioartificial pancreas; CGM, continuous glucose monitor; T1D, type 1 diabetes.

#### Medical Benefits

##### Improved clinical outcomes

Participants expressed hope of gaining improved glycemic control with a BAP. They expected that improved control would affect their short-term well-being, such as having fewer mood swings while also reducing the likelihood of complications in the long run, including the risk of developing chronic kidney disease. Moreover, particularly younger participants hoped that less fluctuating blood glucose levels would decrease the risks of complications during pregnancy.

##### A cure for diabetes

Participants differed in the extent to which they considered a BAP to be a cure for T1D. Whereas some expected that the BAP would make it possible to “live like a person without diabetes,” others argued that the BAP would not restore the function of their pancreas and perceived it as a “nice medical aid” that could relieve some of the burden of their disease.

#### Psychological Benefits

##### More headspace

In the context of managing diabetes, “headspace” refers to the cognitive or mental capacity dedicated to a task. Participants emphasized that with their current treatment, they could never take a break from managing their disease. Most participants expected that with a BAP, it would not be necessary to constantly check their blood glucose levels and think about the choices they have to make around food intake, physical activity, and supplies they have to bring. Participant 19 mentioned that a BAP “would feel like a vacation.” Several participants said that, potentially, this therapy would give them more freedom, time, and mental space to focus on other aspects of their lives, such as eating healthy, doing sports, and investing in relationships.

##### Emotional relief

Participants especially expressed worries about events that can occur at inconvenient times, leading, for instance, to sudden fainting or coma at night, which could endanger themselves and frighten their loved ones. Participants were optimistic that the stress and worries associated with living with T1D could disappear with a BAP, ultimately providing emotional relief, not just for themselves but also for their loved ones. They envisioned having less anxiety about long-term diabetes complications and the occurrence of hypoglycemic or hyperglycemic events.

##### Shift in responsibility

Some participants felt judged or blamed by others, including their treating physicians, when their blood glucose levels were suboptimal. Several participants envisioned that with a BAP, the responsibility for maintaining optimal glucose levels would shift from themselves to the transplanted BAP.

##### Protection of privacy

When people make use of CGM/continuous subcutaneous insulin injection devices, they generate data, such as percentages of time during which the glucose levels fall within specific target ranges. These data are shared with device manufacturers, as well as treating physicians and family members. Participants indicated a preference for a BAP because it does not involve data generation or data sharing.

#### Social Benefits

##### Improved flexibility in daily life

Participants anticipated an improvement in their ability and capacity to (spontaneously) participate in social activities with a BAP. This improvement was expected to result from reduced hindrance caused by disease-related physical complications, self-management, and fear of the occurrence of hypoglycemic events. For example, participant 5 mentioned that she often would not join (unplanned) social events because she was scared of getting high blood glucose levels. She said: “it’s not worth it for me, no matter how fun it is.” Additionally, participants foresaw enhanced participation in work-related activities.

##### Disease being less visible

Participants emphasized that their disease is visible to others when they inject insulin or when they wear a CGM or continuous subcutaneous insulin injection device. They hoped that with a BAP, their diabetes would not be visible and that there would be less stigmatization and fewer negative responses from others.

##### Improved relationships with others

Several participants expressed guilt and embarrassment toward loved ones who had to take care of them during hypo or hyperglycemic events. For example, participant 3 emphasized that her husband “will always be a little anxious” about the occurrence of hypoglycemic events. In addition, several participants mentioned that disease management and mood swings resulting from hypoglycemic events have a negative impact on their (romantic) relationships. Some participants hoped that their relationships with others would improve with a BAP, as their loved ones would have fewer worries and they would have more capacity to take care of or invest in relationships.

### Concerns Related to the BAP and Considerations in Decision-making

Concerns of participants related to the BAP and their considerations in decision-making were found across medical, psychological, and societal-ethical domains (Table 3). These were categorized into 6 subthemes “I would consider it (a BAP transplantation) if...”: it is safe enough, it is functional, the surgery is not too invasive, lifelong follow-up is not too intrusive, the location is acceptable, and I support the biological material used. Table 3 is not intended as a “checklist” because key considerations for one person may not necessarily apply to someone else.

**TABLE 3.** Participants’ concerns and considerations in decision-making about the BAP

Category	I would consider it (a BAP) if	Quote
Medical	It is safe enough - The chance of adverse events is low - It is properly tested in clinical trials - The risks associated with the biological materials used are low - I would not have to take immunosuppressive medication	“… If you introduce modern new technology in your body then it has to be carefully done, especially in people with T1D, who have problems with their autoimmune system, that the product [the BAP] will respond well to your body. They [the researches] will use multiple cell sources, therefore they [the researches] have to be sure that the product [BAP] will not be rejected. I do not want to be in the situation where you would need to use extra medication to prevent rejection. This is an important concern for me.”—participant 3
	It is functional - The benefits outweigh the risks - It doesn’t have to be replaced very often - The chance of success is high - It reduces the burden of disease management	“I would be concerned about how It will function over the years, whether it [the BAP] will take over the function as well as a real pancreas, that your immune system will not reject it [the BAP]…. Because you would not want to have perfect control for the first 10 year, and that you found out 20 years later that it is functioning less and you suddenly get long-term complications from your diabetes, or that suddenly the product [the BAP] will produce too much insulin and you die from a hypoglycemia. Long-term follow up to have frequent checks if it [the BAP] still [functioning] well, will be important.”—participant 21 “It must be a functional improvement compared with what I have now, preferably without getting other complications…it is important to me that I can maintain the freedom that I have now and that it [the BAP] will reduce the current disease related burden.”—participant 16
	The surgery procedure is not too invasive - The healing of the wound is optimal - The anesthesia needed is low - I don’t have to stay too long in the hospital for surgery	“I do not want to say that I am concerned that I will die after the surgical procedure if it goes wrong. That is probably always possible, I am more concerned that you might come out worse than you went in. And that is for me the point of no return, so to speak, you have that uncertainty…. Once you have the product transplanted, will you be back to your old self after a week or will you have to recover for six months…. Suppose you have to recover from the surgery for two weeks and everything works. Then I would say: just do it every year, no problem. If I will spend two weeks per year recovering and you can go on for the rest of the year. It will also give you more freedom. I am willing to do that.”—participant 17
Psychological	Lifelong follow-up is not too intrusive - The frequency of follow-up visits is acceptable to me	“It really depends on the intensity of the follow-up procedure. If it is intrusive, but for a short period of time, or less intensive but for a longer period of time, the rest of your life, that it [the follow-up procedure] will be an important consideration for me. If I need to go to the hospital every week for check-ups then I would not consider this therapy.”—participant 4
	The location is acceptable - It is effective and does not get damaged easily - It is not visible for outsiders - The size and location do not pose a burden in daily life - It is easily accessible to be able to check its graft function	“At least on a place that is not vulnerable, it should be functional, that first of all. Yes, you do not want to have a scar on your face, it should have to be aesthetically pleasing… Preferably not on a visible place and also the feeling that it is safe, that you don’t have to be careful... That you can easily pump in to it. Above all, it must be safe and I think indeed aesthetically pleasing and not uncomfortable. So yeah, I think those are very obvious criteria for me.”—participant 4 “I would prefer to implant it [the BAP] somewhere easily accessible, so that in case something is wrong … that you can easily and quickly remove it and also that you can place it where you can still feel it and can keep an eye on it.”—participant 21
Societal and ethical	I support the biological materials used - Researchers/manufacturers do not use cells derived from deceased donors - Researchers/manufacturers do not use animal cells - If animals are being used, researchers/manufacturers ensure the well-being of the animals - If animals are being used, my religious leaders must agree with the use of this technology	“I am sensitive about sentimental stories of people who have received a heart and then get some kind of characteristic form them. Whether that is true or not, there are parts of someone else in you…. That will feel intrusive to me, because that is not me. It will feel confusing.... That is going a step too far for me. Then I would find a pig much more pleasant. I find that more natural.”—participant 10

BAP, bioartificial pancreas; T1D, type 1 diabetes.

#### Medical

##### It is safe enough

Whereas some participants (notably, those with poorer control or who experience a high mental burden related to diabetes management) were willing to take some risks of potential harm, most were unwilling to accept any risks mostly depending on their satisfaction with their current treatment. They specifically highlighted the need for reliable results from prior clinical trials on safety and risks of BAP rejection, allergic reactions, tumorgenicity, and zoonotic transmissions. In addition, participants voiced concerns regarding leakage of the BAP, fearing interactions of cellular components with the rest of their body, resulting in irreversible damage. Several participants feared an autoimmune response against the BAP similar to that against their own pancreas. Most participants mentioned that they were not willing to accept a BAP if immunosuppressive medication would be necessary, believing that the benefits of the BAP would not outweigh the risks of the medication.

##### It is functional

Participant views on the functionality of the BAP varied mostly depending on their satisfaction with current treatment options, their ability and capacity to maintain stable blood glucose levels, and their experienced psychosocial impact and burden of managing their disease. Several participants feared that the therapy would administer incorrect insulin amounts or the necessity for another transplant if the effectiveness of the BAP deteriorated over time. A few participants foresaw disappointment when they had to revert to their previous treatment after experiencing life without the burdens associated with constant maintenance of blood glucose levels. Some argued for a minimum graft survival of 5 y, whereas others considered a BAP retransplant each year as a successful alternative. In addition, numerous participants mentioned a certain success percentage. Conversely, some explained that they were content with their current treatment and would not want a transplant, even if it functioned flawlessly.

##### The surgery procedure is not too invasive

Most participants were not concerned about the surgical procedure, expecting the product to be small, subcutaneously inserted, and requiring minimal anesthesia. However, some older participants aged between 60 and 90 y expressed concerns about the potential risks associated with undergoing an intervention. For instance, worries about slow wound healing, anesthesia, scar tissue formation, and infections were mentioned. They highlighted that surgical procedures can be particularly concerning for people with T1D because persistent high blood sugar levels reduce blood flow and the supply of oxygen-rich blood to damaged tissue.

#### Psychological

##### Lifelong follow-up is not too intrusive

Participants expected thorough posttransplant monitoring, involving clinicians or researchers tracking their health status and assessing whether the BAP remains intact and functioning as intended. They anticipated that the frequency of hospital visits would be acceptable and decline over the years. All participants were open to lifelong posttransplant follow-up, considering it a time-saving tradeoff for overall diabetes management.

##### The location is acceptable

Participants varied in their preferences regarding the transplantation site, with the site associated with optimal functioning being prioritized. Another commonly mentioned consideration was that the BAP should not disrupt daily activities. In addition, participants highlighted the importance of choosing a site that ensures the protection of the product and minimizes the risk of damage. Concerns about aesthetics and worries about the visibility of the transplant to others were also mentioned. Most participants favored a smaller device. Overall, participants emphasized that the transplant site would not significantly impact their willingness to undergo the treatment.

#### Societal and Ethical

##### I support the biological material used

Most participants did not express any moral objections to using biological materials derived from various sources to generate a BAP. A few participants, however, were averse to receiving islets from deceased donors, preferring pig islets instead. They envisioned that cells from human donors might retain the characteristics of the donor. Moreover, participants stated concerns about the treatment of animals in research, with some making their acceptance of a BAP conditional on the developer’s commitment to consider animal welfare. One participant had religious reservations about using pig islets but still considered it justified for curing diabetes.

### Procedural Considerations

Participant perspectives regarding procedural aspects of clinical research and implementation of the BAP are explained below and summarized in Table 4.

**TABLE 4.** Participants’ perspectives on procedural considerations of clinical research and implementation of the BAP

Category	Procedural aspects	Quote
Trust and control	• Transplanted persons should be able to use CGM devices initially to check blood glucose levels and graft functioning • An emergency plan should be in place • If the BAP fails: - patients should be able to go back to their previous treatment - persons should be able to participate in future research projects - it should be possible to remove it	“… [I]f they would tell me: ‘You don’t have to inject anymore’ then I would be a little upset, because then I would think: ‘Well, that’s not possible, is it?’ Right? It has become a huge part of my life. Look, any other person would put on glasses, and I inject, right? It is just like saying, if you don’t put on your glasses, then you won’t see and if I don’t inject, then it won’t go well either.”—participant 19 “If you undergo a treatment what will make you sick, or you experience certain side-effects, if you know that it can also be removed again. It would be valuable to know that and it can be reassuring for me.”—participant 21
Equitable access	• Access to the BAP should be equal globally • The treatment should be reimbursed	“If it becomes clear that this [BAP] can also be relatively easily deployed in Africa- so not only in rich countries, but everywhere…. If its [the BAP] benefits are high on a global level and if it’s [BAP] affordable … only then I want to be a part of that.”—participant 20
Patient prioritization	• Younger persons should be prioritized	“Yes, maybe I would like to have one [a BAP], but maybe it is better to provide it [the BAP] to younger people. I still have a whole life ahead. Yes, my life is almost over…. For me personally it is not worth it to make the investment, it costs probably a lot of money.”—participant 2

BAP, bioartificial pancreas; CGM, continuous glucose monitor.

#### Trust and Control

Participants often emphasized being reluctant to surrender some of their control and autonomy if they would receive a BAP. They described maintaining control as a significant aspect of their daily routine, which they expect to be difficult to relinquish or change. Once transplanted, without any device to check the blood glucose levels, they will lose insight into their health status. In addition, participants expressed the need to build trust regarding the functionality of the BAP during the initial months posttransplantation, emphasizing the need for a (1) CGM to monitor blood glucose levels and (2) an emergency plan that outlined how to identify and respond to a malfunctioning BAP. Finally, some participants advocated for the possibility of the removal of the product in its entirety.

#### Inequitable Access

Participants often referred to the existing inequalities in access to CGM devices, fostering doubts about equitable access to this potential therapy. They were worried that the high developmental costs of the BAP could make the treatment inaccessible for many, especially for people living in less developed countries. Participant 4, for instance, stated that “high developmental costs would partly contribute to inequality.” Some participants even insisted that equitable access to the BAP should be a condition. For instance, participant 16 emphasized, “it should be available worldwide for all [individuals with T1D] who might need it.”

#### Patient Prioritization

Some argued that younger individuals should be prioritized because they could benefit most from a BAP treatment and enjoy longer lives with fewer diabetes-related complications. In addition, older people (aged 60 y and older) were unsure about whether they would be eligible for BAP treatment because of their age or diabetes-related complications or if they anticipated no longer being alive when the product becomes broadly available. Conversely, a few participants argued for offering the BAP to the older generation of people with T1D first, considering that they have had to endure the disease the longest.

## DISCUSSION

This study reveals that people with T1D consider the BAP as a potential future therapy that may provide not only medical benefits but also psychological and social benefits.

First, they hope the BAP will minimize the psychological burden of self-managing blood glucose levels. With diminishing self-management, the responsibility for maintaining optimal glucose levels will transfer from individuals to the BAP, resulting in “a shift of responsibility” and “more headspace.” Second, diabetes-related fears, such as the occurrence of hypoglycemic events, persist with current diabetes treatment. That these fears can negatively affect the social lives of people with T1D, including social relationships and the ability to participate in social activities and work, is consistent with earlier empirical research.^19^ With the prospect of having more constant blood glucose levels with a BAP, diabetes-specific fears could disappear, explained by people with T1D as ultimately providing “emotional relief.” The dependency and burden on their loved ones could also be reduced, resulting in “improved social relationships,” and the ability and capacity to regularly or spontaneously participate in social activities and events could lead to “improved flexibility in daily life.” Finally, our results indicate that people with T1D generally prefer a small BAP that makes their “disease less visible,” which is in line with findings from previous questionnaire studies.^20,21^ Likewise, other empirical studies showed that people with T1D who wear devices express worries regarding the stigmatization associated with having a disease.^22–25^ The relative invisibility of the BAP compared with other device-based treatments could increase the feeling of “normality” in people with T1D because of fewer negative responses from others.

Our findings indicate a discrepancy between the psychological hoped-for benefits of “more headspace” and “a shift of responsibility” and the psychological concern of “loss of control.” On the one hand, people with T1D expect to have more headspace and feel relieved when they are no longer confronted with blood glucose levels and are the ones bearing the responsibility for controlling their disease. On the other hand, they imagine it to be challenging to no longer be responsible for maintaining control and to entrust disease management to the BAP. This is in line with previous research conducted during the implementation of HCL.^3,25,26^ In these studies, people with T1D were informed by clinicians that the system could only work optimally if they did not interfere with the system and let the algorithm do its work. The study by Taleb et al,^26^ for example, showed that 88% of the participants preferred to ignore or override algorithms. Similarly, other empirical studies indicated that having trust in the accuracy of their device may affect adherence; only once people with T1D trusted the technology could they embrace it.^27^ Likewise, having trust and being in control were frequently mentioned in this interview study. The distinction between advanced devices such as HCL systems and the BAP is that devices can still be manually manipulated, for instance, by administrating additional insulin. However, this manual control will not be possible for therapies that are implanted in the body, like the BAP. This difference highlights why having trust may be even more crucial for people contemplating a BAP transplant. Our findings give insights into how trust can be built: (1) facilitate the use of a CGM device and (2) provide a concrete emergency plan to build trust during the transition period, which may help ensure responsible translation of the BAP from bench to bedside.

Our results showed that most persons with T1D would not consider a BAP transplant if it required daily immunosuppressive medication because they believe that the benefits of the BAP would not outweigh the risks associated with the medication. These findings suggest that any need for such medication could hinder the implementation of the BAP in clinical care. Persons with T1D highlighted this concern, although they had not received detailed information about whether immunosuppressive medication would be necessary or about the associated risks. Thus, this result indicates that participants had some prior knowledge of immunosuppressive risks before the interview. Moreover, results suggest that attitudes toward receiving a BAP varied between older (aged 60 y and older) and younger (aged 20–39 y) people with T1D. Older individuals expressed more hesitation about undergoing potentially invasive therapy. This result is in line with earlier questionnaire studies.^20,21^ These studies also found that younger women with T1D cared significantly more about the visibility of the therapy than older people and that younger people were more likely to accept a shorter functional duration of the graft.^20,21^ In addition to age, perceptions regarding the BAP constituting a cure could influence decision-making. Our findings align with the questionnaire study by Tol et al^21^: one-third of the participants in that study indicated that they would only consider a BAP if it offered a “cure.” Our results suggest that people with T1D have differing perspectives on whether the BAP is a “cure” for T1D. Therefore, their perspectives may influence their decisions regarding treatments and are important to explore when offering education and obtaining informed consent for treatments. Greater clarity is needed on how information about the BAP should be presented so that professionals can responsibly inform people with T1D. Providing accurate information is also important to maintain a relationship founded on trust.

### Strengths and Limitations

This study adds to the requirement of the European Medicines Association to include patients’ voices throughout clinical translation to ensure responsible research and development of this novel therapy.^28^ Furthermore, using a qualitative approach allowed participants more flexibility to express themselves compared with the previously conducted quantitative, closed-ended questionnaire studies.^20,21^ As a result, participants reported psychological, social, and societal benefits and concerns regarding the BAP, which had not been identified in earlier research. Moreover, it may have been difficult for participants to imagine the risks and benefits of a hypothetical therapy. We used a standardized informational process at the beginning of each interview in which we sought to provide sufficient technical detail as well as neutral information on possible risks and benefits so as to not unduly influence participants’ perspectives (see **Supplemental Material 2, SDC,** http://links.lww.com/TXD/A700).

### Future Research

Looking ahead, the perspectives of islet/pancreas transplant candidates and recipients should be explored, because these might differ from the perspectives identified in our sample. These individuals have more (experiential) knowledge about the risks and benefits of transplant-based therapies. Specifically, transplant recipients have experience with immunosuppressive medication and associated lifestyle restrictions, and some may know what it is like to be insulin independent after years of self-management with insulin. In addition, access to BAPs will depend on the knowledge and views of diabetes professionals; however, their perspectives regarding the BAP have yet to be investigated. Such research is important to gain insight into their attitudes and views on potential barriers and facilitators of the clinical implementation of this therapy in the clinic. Finally, conducting a comparative study among subgroups (e.g., those persons with poorer control or those using novel HCL systems) before implementing the BAP would be important to identify specific needs within these groups. We propose using a mixed-method approach, including the use of a questionnaire to allow for quantitative comparisons. The findings of our study, including the identified themes and subthemes, could serve as the groundwork for such a mixed-method study.

## ACKNOWLEDGMENTS

Members of the VANGUARD consortium: Ekaterine Berishvili, Laura Mar Fonseca, Fanny Lebreton, Kevin Bellofatto, Juliette Bignard (Department of Surgery, University of Geneva, Geneva, Switzerland); Jochen Seissler, Leila Wolf-van Buerck, Mohsen Honarpisheh, Yichen Zhang, Yutian Lei, Monika Pehl (Diabetes Centre—Campus Innenstadt, Medizinische Klinik und Poliklinik IV, Klinikum der Ludwig-Maximilians-Universität München, Germany); Antonia Follenzi, Christina Olgasi, Alessia Cucci, Chiara Borsotti, Simone Assanelli (Department of Health Sciences, University of Piemonte Orientale, Novara, Italy); Lorenzo Piemonti, Antonio Citro, Silvia Pellegrini, Cataldo Pignatelli, Francesco Campo (IRCCS Ospedale San Raffaele, Diabetes Research Institute, Milano, Italy); Olivier Thaunat, Morgane Fouché (Department of Transplantation, Nephrology and Clinical Immunology, Lyon Claude Bernard University, Lyon, France); Devi Mey, Chiara Parisotto, Giovanna Rossi (European Society for Organ Transplantation, Padova, Italy); Patrick Kugelmeier, Markus Mühlemann, Karolina Pal-Kutas (Kugelmeiers AG, Erlenbach, Switzerland); Marco Cavallaro, Julia Götz, and Jeanette Müller (Accelopment Switzerland Ltd.).

## Supplementary Material


