# Supplementary material for: Clinical Translation and Implementation of a Bioartificial Pancreas Therapy: A Qualitative Study Exploring the Perspectives of People With Type 1 Diabetes
Source: Transplant Direct. 2024 Sep 25;10(10):e1711. doi: 10.1097/TXD.0000000000001711 (PMC11427030; doi:10.1097/TXD.0000000000001711)
Supplement: Supplementary file 1 [file txd-10-e1711-s001.pdf]

Supplementary Material 1 - Interview guide (translated)

Topic 1: Impact of diabetes on daily life
- How does the diabetes(management) impact your daily life?

Topic 2: Experiences with current available diabetes therapies
- How do you administrate insulin?
- Do you use a glucose monitoring device, and if so, which one?
- What were your considerations in decision-making to choose for your current diabetes therapy?
- What are the pros and the cons of the therapy you use?
- How can your current therapy be improved?

Topic 3: Expectations on bio-artificial pancreas treatment
- What do you personally expect regarding potential advantages and disadvantages of the bio-artificial pancreas compared to alternative treatment options for T1D?
- How could the bio-artificial pancreas potentially impact your daily life?

Topic 4: Attitudes regarding mixing cells
- How do you feel about the fact that, in addition to your own cells, the product may also contain cells from (deceased) donors?
- What is your view on the use of animal cells to generate this product?
- What is your view on using genetically modified cells?

Topic 5: Considerations regarding the location
- Which transplant location do you prefer? Explain your considerations.
- Will the location affect your experience with the treatment?

Topic 6: Informational needs
- What kind of information do you need from researchers regarding the product itself or about the development of the product in order to provide informed consent to receive a bio-artificial pancreas transplantation in the future?
- What do you prefer to know about the surgery procedure?
- Would you consider to participate in a clinical trial? Why? or why not?
- Which risks do you expect regarding a bio-artificial pancreas transplant?
- What can researchers do to decrease your fear regarding the expected risks?
- Are there any other aspects important for you in order to provide informed consent?

Topic 7: Attitudes regarding lifelong monitoring
- How do you view the fact that after transplantation your health have to be monitored by a researcher/physician for the rest of your life?
- What could be possible barriers regarding lifelong monitoring?

- What could researchers/physicians do to make monitoring more bearable for you?

Topic 8: Conditions to consider a bio-artificial pancreas therapy
- What are crucial requirements for you to consider a bio-artificial pancreas treatment?
- When will a bio-artificial pancreas be considered a successful treatment for you?

Original interview guide (in Dutch)

Topic 1: Impact van diabetes op het dagelijks leven
- Wat voor invloed heeft diabetes(management) op uw dagelijkse leven?

Topic 2: Ervaringen huidige behandelmethodes
- Hoe reguleert u uw diabetes? Met of zonder een glucose monitoringsysteem?
- Wat waren voor u overwegingen om voor uw huidige behandeling te kiezen?
- Wat de voor- en nadelen van de behandelmethode die u op dit moment gebruikt?
- Wat zou uw huidige behandeling beter kunnen maken?

Topic 3: Verwachtingen op de bio-artificiële alvleesklier als behandeloptie
- Wat zijn volgens u persoonlijk verwachte voor - en nadelen van de bio-artificiële alvleesklier in vergelijking met alternatieve behandelopties voor diabetes?
- Op welke manier zou een bio-artificiële alvleesklier behandeling het dagelijkse leven van u kunnen beïnvloeden?

Topic 4: Attituden rondom het mixen van cellen
- Hoe staat u ertegenover dat er naast uw eigen cellen ook wellicht cellen afkomst van (overleden) donoren in het product kunnen zitten?
- Wat zijn uw opvattingen tegenover het mogelijk gebruik van dierlijke cellen in het product?
- Wat zijn uw opvattingen over het genetisch modificeren van cellen?

Topic 5: Overwegingen over de plek van het product
- Welke plek heeft uw voorkeur? Leg uit, wat zijn uw overwegingen?
- Maakt de plek van transplantatie uit voor hoe u de behandeling beleeft?

Topic 6: Informatiebehoefte
- Welke informatie heeft u nodig van onderzoekers over het product zelf of over de ontwikkeling van het product om geïnformeerde toestemming te kunnen geven om een bio-artificiële alvleesklier te laten transplanteren in uw lichaam?

- Welke informatie over het plaatsen van het product in uw lichaam zijn voor u belangrijk om geïnformeerde toestemming te kunnen geven om het product te transplanteren?
- Zou u mee willen doen aan een klinische trial? Waarom wel of waarom niet?
- Wat ziet u als mogelijke risico's die kunnen optreden bij een bio-artificiële alvleesklier transplantatie?
- Wat zouden onderzoekers kunnen doen om de angst rondom verwachte risico's kleiner te maken?
- Zijn er nog andere zaken van belang bij het geven van toestemming?

Topic 7: Attitude rondom levenslang monitoren

- Hoe kijkt u aan tegen het feit dat u mogelijk na transplantatie uw hele leven lang gemonitord zou moeten worden door een onderzoeker/arts om uw gezondheid in de gaten te kunnen blijven houden?
- Wat zouden mogelijke barrières voor u kunnen zijn rondom levenslang monitoren?
- Wat zouden onderzoekers/artsen kunnen doen om het monitoren voor u dragelijker te maken?

Topic 8: Voorwaarden om een bio-artificiële alvleesklier behandeling te overwegen

- Waar zou de bio-artificiële alvleesklier aan moeten voldoen om een geschikte alternatieve behandeling te zijn voor u?
- Wanneer is volgens u een behandeling met een bio-artificiële pancreas succesvol?

Supplementary Material 2 - Script of the introduction presentation (translated)

At this moment, researchers working in the lab are developing a tailor-made, immune-protected bio-artificial pancreas for people with T1D. The development of this product is still in its infancy, and not expected to be available soon. What is a bio-artificial pancreas? The research approach involves generating a bio-artificial pancreas from biological material derived from multiple sources. The bio-artificial pancreas might be made out of patient's own cells, so recipients ideally would not have to take immunosuppressive drugs. In addition, the bio-artificial pancreas could contain cells that have been genetically modified to prevent an immune response from the recipient. These cells might be derived from donated placentas. And, the islets of Langerhans that ensure the insulin secretion of the product could be originated from deceased donors or perhaps piglets. Currently, there is a shortage of deceased donors, therefore piglet's islets could be an alternative source. Similar research groups are trying to generate insulin-producing cells derived from induced pluripotent stem cells. Eventually the cells derived from multiple sources will be combined and protected by a scaffold, for example a so-called hydrogel scaffold. The researchers hope that once the bio-artificial pancreas is transplanted into a person with T1D, the individual will no longer need to self-administer insulin, as they

potentially become insulin-independent. In summary, this interview focusses on the development of a personalized, immune-protected bio-artificial pancreas made entirely of biological material, which researchers intend to transplant into patients with type 1 diabetes.

Why do we conduct this interview study? That developers can take patients preferences, needs and wishes into account in the design of the treatment and development. This ensures that once this treatment will be tested in clinical trials or eventually becomes available and accessible for more persons with T1D, their needs are taken into account. For instance, researchers do not yet know where the product will be placed in the body. What are the needs of persons with T1D regarding a transplantation location? Does this novel approach offer a desirable alternative compared to the treatments currently available for people with T1D? What do patients expect as potential risks when this product is transplanted into their body? These are all questions I am going to ask you in the next hour.

Script in Dutch

Op dit moment zijn lab onderzoekers bezig met de ontwikkeling van een op maat gemaakte bio-artificiële alvleesklier voor personen met T1D. De ontwikkeling van dit product staat nog in de kinderschoenen, dus de mogelijke therapie voor T1D waarover ik het heb is echt nog toekomstmuziek. Waar bestaat een bio-artificiële alvleesklier uit? Het idee is dat de bio-artificiële alvleesklier geheel wordt gemaakt van biologisch materiaal en bestaat uit verschillende type cellen met wellicht een andere oorsprong. In de bio-artificiële alvleesklier kunnen mogelijk patiënt eigen cellen in zitten, waardoor de patiënten ideaal gezien geen afweermedicijnen hoeven te slikken. Daarnaast kunnen er cellen worden gebruikt die genetische gemodificeerd zijn, zodat er geen immuunreactie kan optreden bij het getransplanteerde persoon. Deze cellen zijn mogelijk afkomstig van gedoneerde placenta’s. En, er zitten eilandjes van Langermans in die zorgen voor de insuline-productie van het product, potentieel afkomstig van overleden donoren of wellicht van varkens. Er is op dit moment een tekort aan overleden donoren waardoor er wellicht gebruikt gemaakt gaat worden van varkenseilandjes. Ook zijn er andere onderzoekgroepen bezig om insuline-producerende cellen te maken vanuit stamcellen. Uiteindelijk worden al deze verschillende cellen met een andere oorsprong samengevoegd, en stoppen onderzoekers de cellen in een omhulsel (scaffold), bijvoorbeeld een hydrogel scaffold. Dit omhulsel zorgt ervoor dat de cellen die de bio-artificiële alvleesklier vormen beschermd worden in het lichaam. Onderzoekers hopen dat wanneer de bio-artificiële alvleesklier is getransplanteerd in een diabetespatiënt zij zelf geen insuline meer hoeven toe te dienen, omdat zij potentieel insuline-independent zijn geworden. Samengevat, het interview gaat dus over de ontwikkeling van een gepersonaliseerde, afweer-beschermde bio-artificiële alvleesklier die onderzoekers willen transplanteren in patiënten met T1D, en volledig bestaat uit biologisch materiaal.

Waarom voeren we dit interview uit? Om alvast naar de voorkeuren van patiënten te vragen, zodat onderzoekers rekening kunnen houden met de behoeften en wensen van een patiënt in het ontwerp van de behandeling. Zo kan er bijvoorbeeld voorkomen worden dat de behoeften van personen met T1D niet worden meegenomen wanneer deze behandeling wordt getest in klinische trials of uiteindelijk beschikbaar komt voor meerdere patiënten. De onderzoekers weten bijvoorbeeld nog niet waar het product in het lichaam wordt geplaats. Wat zijn de behoeften van T1D patiënten als het gaat om een geschikte plek? Biedt deze mogelijk behandeling een alternatief in vergelijking met de behandelingen die nu beschikbaar zijn voor mensen met T1D? Wat verwachten patiënten als mogelijke risico’s wanneer dit product wordt getransplanteerd in hun lichaam? Dit zijn allemaal vragen die ik u het komende uur ga stellen.
